# A Benevolent Enterprise

**Published:** 1855-05

**Authors:** 


### A BENEVOLENT ENTERPRISE.

The highly gifted Bayard Taylor, in traveling through Palestine, describes many interesting features of the country, and various incidents connected with his travels, which he gives in his glowing letters. Among the ancient and *peculiar* institutions of that land, he places the following in the *cat*-egory and *prominent* among the others. This may prove a curiosity to our medical friends who would doubtless be much gratified with the character of the clinical instruction to be gleaned within its walls. He describes it as follows :

" HOSPITAL FOR CATS AT ALEPPO.—The other remarkable thing here is the hospital for cats. This was founded long ago by a rich, cat-loving Mussulman, and is one of the best endowed institutions in the city. An old Mosque is appropriated to the purpose, under the charge of several directors ; and here sick cats are nursed, homeless cats find shelter, and decrept cats gratefully purr away their declining years. The whole category embraces several hundreds, and it is quite a sight to behold the court, the corridors and terraces of the Mosque swarming with them. Here, one with a bruised limb is receiving a *cat*aplasm ; there, a *cat*eleptic patient is tenderly cared for ; and so on, through the long con*cat*enation of feline diseases. Aleppo, moreover, rejoices in a greater number of cats than ever Jerusalem. At a rough guess, I should thus state the population of the city: Turks and Arabs, 70,000 ; Christians of all denominations, 15,000 ; Jews, 10,000 ; dogs, 12,000, and cats, 8,000."

